# Composite proteomic and metabolomic plasma biomarkers for detection of colorectal, lung and ovarian cancers

**DOI:** 10.1186/s12943-026-02654-1

**Published:** 2026-04-07

**Authors:** Jim Åkerrén Ögren, Joakim Ekström, Natallia Rameika, Emma Torell, Chatarina Larsson, Ivaylo Stoimenov, Patrick Micke, Ulf Gyllensten, Mats Hellström, Bengt Glimelius, Karin Stålberg, Tobias Sjöblom

**Affiliations:** 1 Department of Immunology, Genetics and Pathology, Science for Life Laboratory, Uppsala University, Uppsala, Sweden; 2 Department of Women’s and Children’s Health, Uppsala University, Uppsala, Sweden

**Keywords:** Colorectal cancer, Lung cancer, Ovarian cancer, Early detection, Plasma biomarkers, Affinity proteomics, ROC AUC, Cancer diagnostics

## Abstract

**Background:**

Sensitive and specific blood biomarkers for early detection of colorectal (CRC), lung (LuCa) and ovarian (OvCa) cancers are highly warranted. The current blood tests often need to be complemented with other clinical methods to achieve adequate diagnostic performance. Recent progress in the molecular profiling of plasma from cancer patients holds potential for improved non-invasive screening. Here, we aimed to identify composite proteomic and metabolomic plasma biomarkers for early cancer detection with performances that exceed those of existing FDA-approved blood and stool-based diagnostic tests for CRC, LuCa and OvCa.

**Methods:**

In a case-control study using samples from the U-CAN and EpiHealth biobanks, we measured plasma levels of 165 proteins and 244 metabolites in 818 patients with CRC, LuCa and OvCa at diagnosis, 119 patients with non-malignant conditions of the corresponding organs, and 1,129 healthy individuals. We performed an exhaustive search over all cut-off values of the ROC for all combinations of up to 4 proteins and metabolites, implementing measures to minimize the impact of cross-cohort comparisons. Ultimately, we benchmarked candidate biomarkers performance to FDA approved blood tests in clinical use for detection of cancer. External validation was performed using publicly available datasets.

**Results:**

We found biomarkers composed of 2–4 proteins separating cases of each tumor type from healthy controls with ROC AUC, respectively for CRC: CEACAM5, FLT1, IL19, Ferritin (AUC 0.89), LuCa: FNDC5, MDK, PLAUR, CEACAM5 (AUC 0.91) and OvCa: MUC16/CA125, PLG (AUC 0.97). The diagnostic performance of these biomarkers was comparable to, and in some instances surpassed, the performance of established tests, such as Epi proColon (AUC 0.82) and Cologuard (AUC 0.93). Metabolites were informative for tumor stage discrimination, especially in LuCa and OvCa. External validation in the CancerSeek dataset showed strong agreement in biomarker performance.

**Conclusions:**

The composite protein biomarkers identified in this study represent a novel opportunity for detection, staging and differential diagnosis of common tumor types. Metabolites are more relevant for tumor staging than early detection. A limited number of well-performing analytes enables cost-effective implementation in clinical diagnostics and merits further evaluation.

**Supplementary Information:**

The online version contains supplementary material available at 10.1186/s12943-026-02654-1.

## Introduction

Colorectal cancer (CRC), lung cancer (LuCa) and ovarian cancer (OvCa) are among the cancers with the highest global incidence, with LuCa and CRC placing 1st and 3rd for all cancers, and OvCa being the 8th most diagnosed cancer in women [1]. Lung cancer and CRC are also the two leading causes of cancer-related death, accounting for 18.7% and 9.3% of all deaths from cancer, respectively, and in females OvCa places 8th with 4.8% of deaths [1]. In most cancer types, stage at diagnosis is an important predictor of survival [2–4]. Diagnosing the tumor at an early stage is generally perceived to increase access to curative surgery for the primary solid tumor, but in e.g. the US, nearly one-quarter of CRC, half of LuCa and up to 70% of OvCa cases are currently diagnosed at a late stage with distant metastases present [3]. Screening programs may contribute to early detection and diagnosis at earlier stage [5] before development of symptoms, and reduce the mortality in CRC [6] and LuCa [7, 8]. In combination with ultrasound, CA125 as a single biomarker was recently found insufficient to support OvCa screening in an asymptomatic population as it did not significantly lower the mortality although more cancers were diagnosed at lower stages [9]. The symptoms associated with CRC, LuCa, OvCa and several other malignancies are often non-specific and partially overlapping at earlier stages, including fatigue, weight loss, abdominal pain, constipation or diarrhea. There is therefore a need for sensitive and specific blood tests that detect these tumor types at early stages and discriminate them from each other.

Circulating biomarkers offer potential for non-invasive diagnostic and screening tools in cancer. Blood testing is safe and associated with high patient adherence compared to other methods such as radiology, endoscopy or stool-based tests [10]. Current large-scale molecular analysis techniques enable parallel measurement of thousands of analytes in small volumes of blood, including e.g. levels, variants and epigenetic or post-translational modifications of proteins, nucleic acids and metabolites. Recent advances in pan-cancer blood biomarker tests include combined detection of proteins with mutational analysis of ctDNA [11] and analysis of cfDNA methylation with/without fragmentation [12–15]. These efforts have led to prospective clinical trials that compare cancer incidence rates in conventional screening methods with blood-based screening tests, exemplified by the NHS-Galleri Trial [16], The PREEMPT CRC study [17], PATHFINDER 2 (NCT05155605), STRIVE (NCT03085888), and SUMMIT (NCT03934866) studies. While the specificity of methylation-based tests is high, sensitivity is lower particularly in early tumor stages for several common tumor types, potentially because of low levels of released ctDNA.

To ensure efficient translation, biomarker discovery should ideally align with the efficacy requirements for diagnostic tests from regulatory authorities, such as the US Food and Drug Administration (FDA). In practice, for a novel biomarker to gain regulatory approval as a medical device for clinical use, non-inferior ROC AUC to an existing approved test for the same purpose must be demonstrated. Diagnostic blood biomarkers of cancer currently in clinical use generally suffer from low sensitivity and/or specificity, which limits their utility. In CRC, blood tests could improve participation rates in screening programs that are currently based on fecal samples [18, 19], however the current best FDA approved blood test to discover CRC, Epi proColon (ROC AUC 0.82) [20, 21], does not reach the performance of either the fecal occult blood test (FIT) alone (ROC AUC 0.88) [22, 23], or the multi-target stool DNA test Cologuard (ROC AUC 0.93) [24, 25]. Notably, across all other cancer types, no blood test with performance comparable to Epi proColon for CRC is currently FDA approved for early detection. In summary, there is a need for robust blood biomarker tests for cancer with diagnostic performance exceeding state-of-the-art.

Here, we analyze plasma proteome and metabolome data using a ROC-based biomarker discovery approach with benchmarking to regulatory approved early detection tests for cancers to find composite diagnostic plasma biomarkers that have suitable properties for further development. The ROC-based framework is broadly applicable to quantitative omics data for which features can be meaningfully ranked. Although metabolomics contributed less to early diagnostic biomarker discovery in the present study, the integration of affinity proteomics and NMR-based metabolomics within a unified statistical framework is demonstrated. This approach is readily extendable to larger, more diverse datasets and additional modalities in the multi-omics space.

## Methods

### Cases and controls

CRC, LuCa, and OvCa were selected as clinically important and biologically distinct malignancies with available harmonized cohorts, enabling evaluation of the proposed biomarker discovery framework across different cancer types. While arising from different organ systems, these cancers originate from epithelial tissues and enable assessment across biologically distinct yet related solid cancers. While parts of the analyses were aimed at identifying pan-cancer biomarkers, the top-performing biomarkers by AUC estimates were tumor-type specific.

The study size was determined according to [26]. Cancer cases were selected from the full population of individuals included 2010–2020 in the longitudinal prospective cohort U-CAN [27] at Uppsala University Hospital. Inclusion criteria for this study for cancer cases included sample freezing time ≤ 4 h after blood draw, registration in the national quality registry for the respective cancer diagnosis (the Swedish Colorectal Cancer Registry, the Swedish National Quality Registry for Lung Cancer, and the Swedish Quality register for Gynecologic Cancer), age at diagnosis range 45–75 (CRC and LuCa) or 40–80 (OvCa); no cancer treatment or surgical intervention before blood sampling; sampling within 90 days of the diagnosis date stated in the respective national quality registry; no known concurrent or prior malignancy reported in the Swedish Cancer Registry; and availability of sufficient plasma volume. Epithelial borderline ovarian tumors (*n* = 24) were included among the OvCa cases. As technical controls for pre-analytical handling, we used plasma samples from patients in U-CAN with non-malignant or pre-malignant conditions in the respective organ sampled under identical handling conditions as the cancer cases. Common benign conditions were colorectal adenomas and polyps, prolapse and incontinence; ovarian cystadenomas of various histology, endometriosis, endometrial hyperplasia and polyps, myomas and cervical intraepithelial neoplasia (CIN); chronical inflammatory lung conditions. The EDTA plasma samples from U-CAN participants had been drawn non-fasting at diagnosis before administration of any anti-cancer treatment, and subjected to central standardized handling at RT followed by freezing for biobanking at -80 °C. Population controls were selected from individuals included in the prospective study EpiHealth [28] in 2010–2015, that had not been registered in the Swedish Cancer Registry as having any type of cancer up to 5 years after sampling. The EpiHealth individuals had been sampled for blood at a test center at Uppsala University following 6 h fasting, with plasma centrifuged and stored for up to 6 h at + 4 °C followed by freezing for biobanking at -80 °C. Although sample handling protocols were similar across collection centers, non-cancer controls processed under identical conditions as the cancer cases were included to mitigate potential center-related variation and to provide bridging. By requiring that candidate biomarkers demonstrate discriminatory performance against both non-cancer controls and population controls, we aimed to reduce the likelihood that observed signals reflect cohort-specific or handling-related effects rather than disease biology.

### Measurements of proteins and metabolites

Plasma levels of 165 different proteins were measured using a suspension array ELISA system (Bio-Plex 200, Bio-Rad) and 10 antibody panels (Human Circulating Cancer Biomarker Panel 1, Human Circulating Cancer Biomarker Panel 3, Human Circulating Cancer Biomarker Panel 4, Human Cancer metastasis Biomarkers panel, Human Immuno-Oncology Checkpoint Protein Panel 1, Angiogenesis Panel 2, Human Cytokine/Chemokine Panel, Human Cytokine/Chemokine Panel II, Human Cytokine/Chemokine Panel IV, Human Myokine Panel) according to the manufacturer’s instructions. Using xPONENT^®^ software (ThermoFisher), data was collected from 50 beads per bead-set for all 10 kits. The OvCa marker WFDC2 (HE4) was measured but excluded for technical reasons (assay failure) after consulting the manufacturer. In the Bio-Plex 200 platform, which is CE-marked for IVD in Europe, the batch effects were minimized through routine calibration using instrument-specific calibration curves performed for each analytical run. The plasma levels of 244 metabolite parameters were determined by NMR using the Blood analysis service by Nightingale Health, Helsinki, Finland. EDTA plasma samples stored at − 80 °C were analyzed according to Nightingale Health’s standardized protocols, including automated spectral processing and absolute quantification. Proprietary software performed spectral processing including phase and baseline correction, peak alignment, and metabolite quantification using calibrated reference standards. Quality control procedures included internal control samples and blind duplicates to monitor instrument performance and consistency across batches.

### Data analysis

For all analytes we computed ROC AUC for distinguishing cancer from non-cancer controls and cancer from healthy controls. Based on extensive study design simulations, all data was used for validation [26]. All data points were included in ROC computations and statistical analyses, and missing data values were internally represented as not-a-number (NaN). Candidate biomarker combinations were evaluated only on samples with available measurements, resulting in case-wise exclusion of missing observations and a corresponding reduction in effective sample size for the analytes comprising the unitary or composite putative biomarkers. In boxplot visualizations, the whiskers extend to 1.5 times the interquartile range, and outliers are not shown due to distortion of scales. Statistical significance at the 95% confidence level was tested using two-sided Mann-Whitney U test and Bonferroni correction for multiple hypothesis testing. The number of hypotheses tested and corrected for was 409*3 = 1227. Analytes that were significant both versus non-cancer controls and healthy controls were included in computation of combination biomarkers. Data analysis and visualization were done in Python and corresponding data science packages (*Python 3.9.18*,* numpy 1.21.4*,* pandas 1.3.4*,* matplotlib 3.5.0*,* seaborn 0.13.0*,* plotly 5.6.0*,* scikit-learn 1.2.1*,* networkx 3.1*). Plots were generated using Python and figure panels composed with Inkscape and biorender.com.

### Composite biomarkers for cancer detection

We used a novel algorithm to compute the ROC curves for combinations of up to 4 analytes while optimizing the cut-off values for each analyte [29]. For a combination of *k* analytes, we computed *2*^*k + 1*^ classification scenarios: each analyte can be up- or downregulated and combined using logical AND or logical OR. For each classification scenario exhaustive search was used to check all possible cutoff values and optimize the ROC curve, i.e. finding highest possible true positive rate for every false positive rate yielding the highest possible AUC. We designed the study according to results from prior simulations in this statistical framework that revealed that the most efficient use of cases and controls given the number of hypotheses tested was to assign all to a validation step [26]. We computed the ROC AUC for separating cancer cases of stages I-IV from the healthy controls of the population cohort given the over/under rules identified in the first step but allowing for optimization of new cut-off values. We then performed statistical hypothesis testing of whether the biomarker separated cases from controls better than a benchmark biomarker with the same properties as the currently best FDA approved diagnostic blood test for cancer, Epi proColon (ROC AUC 0.82), and the two best feces-based tests Fecal Immunochemical Test FIT (ROC AUC 0.88) or Cologuard (ROC AUC 0.93) at the 99% confidence level given the same over/under rules that were generated in the comparison of cancers to non-malignant disease controls. This process was repeated for each tumor type. We used Bonferroni correction to adjust p-values and corrected for all computed combinations of respective order. Thus, we evaluated in total 708 2-combinations, 3,892 3-combinations and 16,480 4-combinations. Composite biomarkers were limited to combinations of two to four analytes to balance statistical power, interpretability, and translational feasibility under strict multiple-testing control; larger panels were not expected to yield robust gains given the available sample size [26].

### Biomarkers for stage separation

The correlation analysis was performed using two-sided Spearman rank correlation test and Bonferroni correction for multiple hypothesis testing (409 hypotheses tested) to find analytes that were statistically significantly correlated with stage. Two-sided Mann-Whitney U test and Bonferroni correction were used to find individual analytes that could discriminate later stages from earlier stages: stage IV vs. stages I-III and stages III-IV vs. stages I-II. From the significant analytes we computed composite biomarkers in the same way as described above.

### External validation of protein biomarkers

For external validation of protein biomarkers, we computed ROC AUC for combinations using the same rule set as above and data from the CancerSeek study, which contains 388 CRC, 104 lung, and 54 OvCa of stages I-III along with 812 healthy controls sampled in the same pre-analytical handling [11]. For the proteins measured in both studies we used the same methodology and performed the same analysis as described above. First, we identified analytes that were statistically significant, computed all possible 4-combination composite biomarkers and compared performance in the two datasets. When computing the composite biomarkers, the cutoffs were optimized in the two datasets separately. This means that the same behavior (up/down-regulation) and combination of proteins were compared but not absolute cutoff values. This is to circumvent baseline differences due to differences in experimental protocol and sample handling.

## Results

### Plasma proteomic and metabolomic analyses of cases and controls

**Fig. 1:**
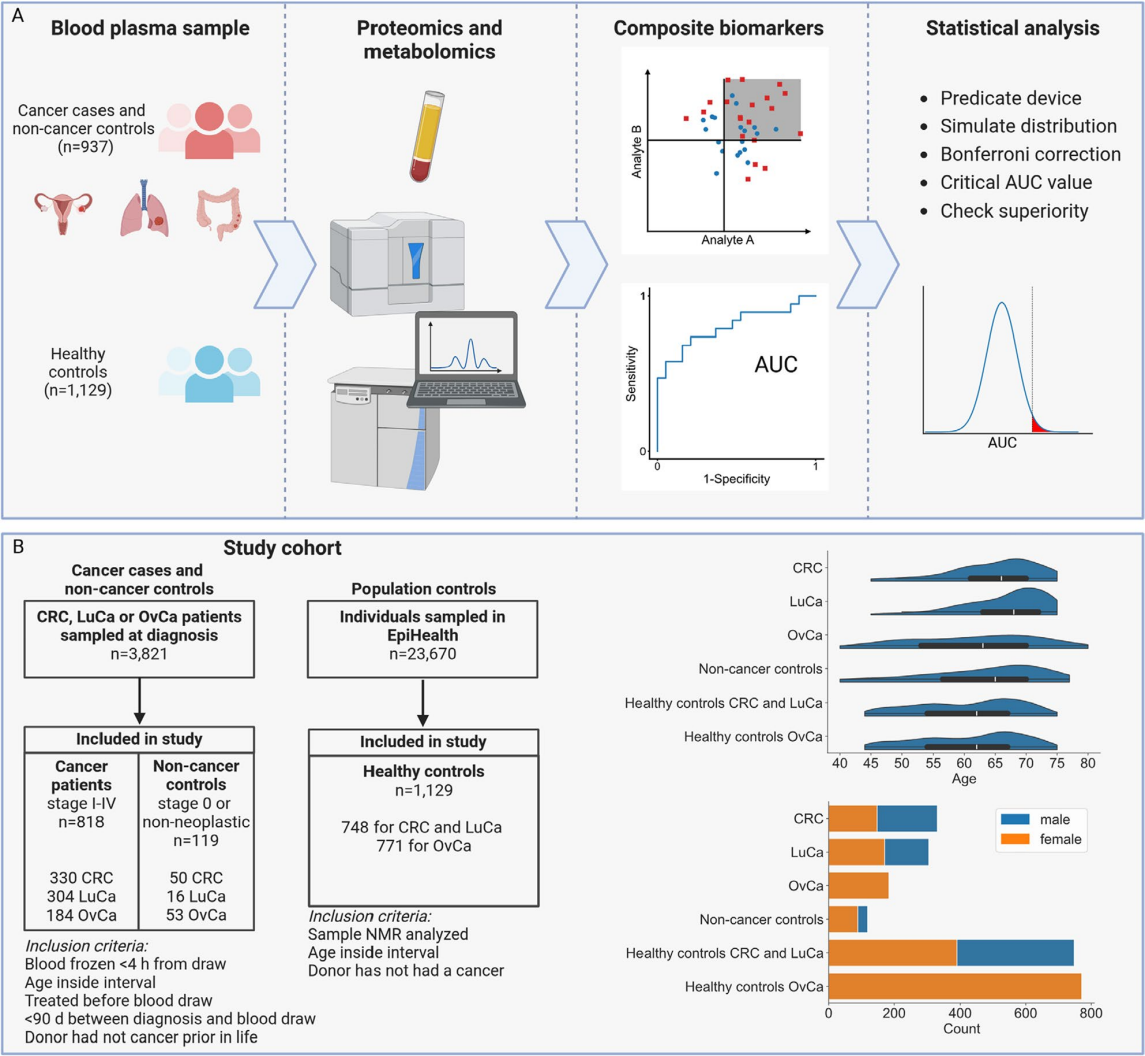
Discovery of tumor-type specific and pan-cancer biomarkers in blood plasma. **A** Plasma samples from patients with cancer or non-malignant conditions at diagnosis and healthy population controls were analyzed by affinity proteomics and NMR metabolomics. The ROC was computed for individual analytes and for composite biomarkers of 1–4 analytes within and across the study cohorts. The ROC AUC distributions for biomarkers with performance similar to a predicate device already in clinical use were used for statistical testing of putative biomarkers. **B** Selection of cases and controls and characteristics of the selected study populations. From 3,821 cases being investigated for colorectal, lung and ovarian cancer, sampled for blood at time of diagnosis in the population-based prospective study U-CAN, 818 with confirmed cancer and 119 with pre-malignant tumors or other disease of the respective organs fulfilled the inclusion criteria. From 23,670 healthy individuals in the population-based cohort EpiHealth, 748, and 771 controls were selected for the CRC and LuCa, and the OvCa cohorts, respectively

To identify diagnostic biomarkers, we measured the levels of 165 proteins and 244 metabolite parameters in plasma samples drawn at diagnosis before any treatment from 330 CRC, 304 LuCa, and 184 OvCa (including 24 borderline tumor) patients with tumor stages I-IV from the prospective population-based cancer biobank U-CAN [27] (Fig. 1A, Supplementary Fig. 1). We also analyzed blood plasma from 119 (50 CRC, 16 LuCa and 53 OvCa) patients with non-malignant diagnosis from the same biobank, here termed Non-cancer controls, to enable selection of biomarkers that discriminate cancer from pre-malignant or non-malignant diseases of the same organs (Fig. 1B, Supplementary Fig. 2). As healthy population controls, we analyzed plasma samples from 1,129 individuals of the EpiHealth cohort [28] that were in the same age span as the cancer cases and living in the same area (Fig. 1B, Supplementary Fig. 1). To ensure a relevant age interval for cancer screening, the cases and controls were of age 45–75 in CRC and LuCa and 40–80 in OvCa. The age and sex distributions of cases and population controls were similar (Table 1; Fig. 1B). Comparing the study cohort to the incident population as recorded in the national quality registries for the respective cancers, we found that for CRC, the distributions of patient sex, tumor location, T and N stage was similar to the incident population (Supplementary Fig. 3). In LuCa, the proportions of smokers, sex and histological subtype were similar, whereas stage I was overrepresented and stage IV underrepresented compared to the incident population (Supplementary Fig. 4). For OvCa, the relative proportion of sub-diagnoses differed, and stage I tumors were underrepresented compared to the population, but there was a similar ratio of stages I-III to IV/X0 (Supplementary Fig. 5). The complete analysis workflow can be divided into two principal pipelines representing the discovery and validation phases of the study (Supplementary Fig. 6). Together, the population-based study cohorts are representative of the incident cancer cases and the background population.

**Table 1:** Characteristics of cancer cases and controls

Characteristic	Cancer cases at diagnosis^a^			Controls		
	CRC	LuCa	OvCa	Non-cancer	Population for CRC and LuCa^b^	Population for OvCa^b^
Cohort	U-CAN	U-CAN	U-CAN	U-CAN	EpiHealth	EpiHealth
n	330	304	184	119	748	771
Female	148 (45%)	171 (56%)	184 (100%)	89 (75%)	390 (52%)	771 (100%)
Average age, years (Std Dev)	64.8 (7.1)	66.8 (6.4)	61.7 (10.2)	63.1 (9.5)	60.4 (8.4)	60.7 (8.4)
Stage I	31 (9%)	122 (40%)	37 (20%)			
Stage II	90 (27%)	32 (11%)	16 (9%)			
Stage III	151 (46%)	59 (19%)	84 (46%)			
Stage IV	58 (18%)	91 (30%)	47 (26%)			

^a^Patients at diagnosis of colorectal (CRC), lung (LuCa) and ovarian (OvCa) cancers, with blood sampled before treatment

^b^Population control groups with sex and age distributions matched to the cases. The female population controls in the CRC and LuCa group were also included in the population control group for OvCa

### Protein and metabolite levels altered in cancer patients versus non-cancer controls

**Fig. 2:**
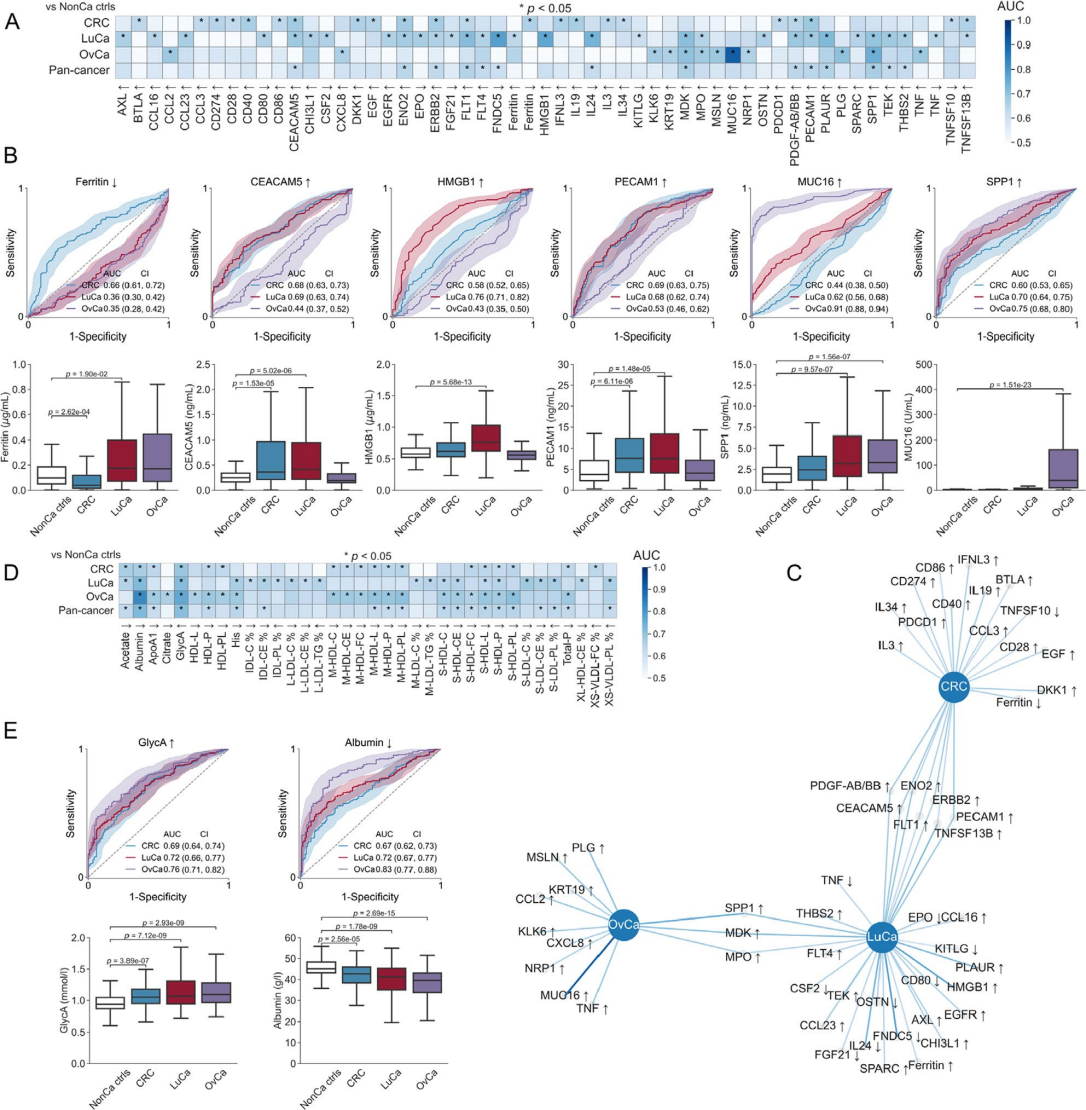
Pan-cancer and tumor-type-specific protein and metabolite biomarkers. For each cancer type alone and together (“pan-cancer”), ROC AUC values were computed for all 165 proteins and 244 metabolites comparing cancer cases against 119 non-cancer controls within the U-CAN cohort. **A** ROC AUC values for proteins significantly up- or down-regulated for at least one cancer type. *denotes analytes at the 95% confidence level (*p* < 0.05). **B** ROC and Box plots for down-regulated Ferritin in CRC, up-regulated CEACAM5 (CEA) in CRC and LuCa, up-regulated HMBG1 in LuCa, up-regulated PECAM1 in both CRC and LuCa, up-regulated SPP1 (Osteopontin) in LuCa and OvCa, and up-regulated MUC16 (CA-125) in OvCa. All *p* values adjusted using two-sided Mann-Whitney *U* test under Bonferroni correction for multiple hypothesis testing. Confidence bands in ROC plots contain 95% of ROC curves from 1,000 bootstrap iterations. **C** Graph visualization of proteins regulated at 95% confidence level. **D** ROC AUC values for metabolites significantly up- or down-regulated for at least one cancer type. **E **ROC and Box plots for pan-cancer markers up-regulated GlycA and down-regulated albumin

Since the cancer cases and healthy population controls were from different biobank cohorts, we saw a risk that pre-analytical effects could affect biomarker identification. Indeed, we observed that the vast majority of NMR analytes differed between non-cancer controls and healthy controls, whereas only a subset of proteins did. In an attempt to minimize the impact of pre-analytical variation, we first analyzed the cancer cases versus the non-cancer controls from the U-CAN cohort, before proceeding to comparing cancer cases to the population controls (Supplementary Fig. 6). First, we computed the ROC AUC for each measured protein as single biomarker in discriminating cancer cases from the 119 non-cancer U-CAN controls and tested for statistical significance (Fig. 2A). From the 165 proteins measured, 22 (CRC), 31 (LuCa), 12 (OvCa) and 14 (pan-cancer) were up- or downregulated in plasma from the cancer patients at diagnosis (*p* < 0.05, 2-sided Mann-Whitney *U* test under Bonferroni correction; Supplementary Tables 1–5). Several established biomarkers for each tumor type were corroborated, including (1) ferritin down-regulation in CRC (AUC 0.66) [30, 31], (2) carcinoembryonic antigen (CEA/CEACAM5) up-regulation with a ROC AUC of 0.68 and 0.69 for CRC and LuCa, respectively, congruent with AUC 0.67–0.80 for CRC [11, 32–39] and 0.62–0.82 for LuCa [11, 40, 41], (3) HMGB1 up-regulation in LuCa (AUC 0.76) [42], (4) SPP1/OPN up-regulation in all three tumor types [11], and (5) Mucin 16 (CA125) with ROC AUC 0.91 for OvCa, in the reported range 0.77–0.97 [11, 43–50] (Fig. 2B). Further, novel potential biomarkers were discovered, including PECAM1/CD31 up-regulation in CRC (AUC 0.69) and LuCa (AUC 0.68). Investigating the overlap between diagnoses, we found that seven significantly regulated proteins connected CRC and LuCa, 3 connected LuCa and OvCa, but none connected CRC and OvCa (Fig. 2C).

Next, we computed the ROC AUC for each NMR determined analyte in discriminating cancer cases from non-cancer controls from the same cohort and tested for statistical significance. From the 244 determined metabolites, 18 (CRC), 21 (LuCa), 21 (OvCa) and 20 (pan-cancer) were significantly up- or downregulated in cancers (*p* < 0.05, 2-sided Mann-Whitney *U* test under Bonferroni correction; Fig. 2D, Supplementary Tables 1–5). Several of these were supported by previous studies. Reduced albumin [51], increased levels of the systemic inflammation marker glycoprotein acetyls (GlycA) [52, 53] along with reduced S-HDL-L and S-HDL-P were potential biomarkers of all three tumor types (Fig. 2E). The CRC and OvCa groups had reduced lipoprotein particles and ApoA1 [54] as well as reduced concentration and altered composition of HDL particles, along with reduced total concentrations of lipoprotein particles (Total-P), phospholipids (Total PL), and free cholesterol (Total FC). The LuCa cases had altered LDL composition with reduced cholesteryl esters and cholesterol fraction and increased phospholipids fraction.

### Composite biomarkers for separation of cancer patients at diagnosis from healthy population controls

We next proceeded to identify composite biomarkers that could discriminate cancer cases from healthy population controls. To remove false positive biomarkers arising from pre-analytical differences between the cohorts, we included only the proteins and metabolites that by themselves could discriminate the cancer cases from the non-cancer controls (Fig. 2A and D). We here required these individual analytes to also have significant difference versus the population controls. Finally, to further ensure that we filtered analytes with large pre-analytical or cohort effects, we retained only those where ROC AUC for cancer vs. non-cancer controls was greater than ROC AUC for non-cancer controls vs. healthy population controls. This resulted in 10 analytes (10 proteins, 0 metabolites) for CRC, 22 (21 proteins and 1 metabolite) for LuCa, and 13 (12 proteins and 1 metabolite) for OvCa identified as suitable for being part of composite biomarkers for the respective cancer type (Table 2, Supplementary Tables 6–9, Supplementary Fig. 6).

**Table 2:** Protein and metabolite analytes with statistically significantly altered levels in blood plasma from cancer cases compared to controls

Analytes	Colorectal cancer	Lung cancer	Ovarian cancer
*vs. Non-cancer ctrls*			
Measured	409	409	409
Significant	40	52	33
*vs. Healthy ctrls*			
Measured	409	409	409
Significant	312	292	292
*vs. Non-cancer ctrls AND Healthy ctrls*			
Significant^a^	24	27	14
Significant after removal of high pre-analytical variation^b^	10	22	13
Remaining upregulated analytes	IL19, CEACAM5, PDGF-AB/BB, FLT1, TNFSF13B, CCL3, IL3, DKK1, IFNL3	PLAUR, FLT1, PDGF-AB/BB, TEK, THBS2, MDK, AXL, CEACAM5, SPARC, FLT4, SPP1, MPO, CCL16, CCL23, TNFSF13B, EGFR, HMGB1, Ferritin, S-LDL-PL %	MUC16, PLG, SPP1, MPO, TNF, MDK, MSLN, KLK6, CCL2, KRT19, NRP1, CXCL8, GlycA
Remaining downregulated analytes	Ferritin	FNDC5, IL24, KITLG	

^a^To limit the impact of pre-analytical differences in the cross-cohort analyses, we required informative analytes to be significant both in comparison to non-cancer controls from the same cohort as the cases and to healthy population controls from a different cohort (analytes at the 95% confidence level, Mann-Whitney *U* test under Bonferroni correction)

^b^Analytes with higher ROC AUC discriminating non-cancer controls from healthy controls compared to ROC AUC discriminating cancer from non-cancer controls were considered as having large pre-analytical effects and were removed from computation of composite biomarkers for cases vs. healthy controls

When forming composite biomarkers for each tumor type, we evaluated each analyte up- or downregulated and all analytes combined using logical AND or logical OR resulting in *2*^*k + 1*^ classification scenarios where *k* denotes the number of analytes combined [26]. For each classification scenario we performed exhaustive search on all possible combinations of cutoff values to obtain the optimum ROC performance, i.e. finding the best true positive rate for each false positive rate. It can be argued that only biomarkers that supersede existing regulatory authority approved and clinically used diagnostic tests are meaningful to discover. We therefore evaluated all combinations of 2–4 up- or downregulated analytes, computed the ROC AUC for different classification scenarios and performed statistical hypothesis testing for superiority versus biomarkers with comparable performance to regulatory approved diagnostic tests. For benchmarking purposes, we used the CRC tests Epi proColon/ColoHealth (AUC 0.82), FIT (AUC 0.88) and Cologuard (AUC 0.93) [29] throughout, arguing that performance of AUC 0.82 is a minimal performance target al.so for early detection of OvCa or LuCa. The FIT and Cologuard tests are feces-based, but they represent state-of-the-art tests for early detection of cancer with performance levels that new biomarkers will need to match or supersede for regulatory approval and adoption in clinical use.

**Fig. 3:**
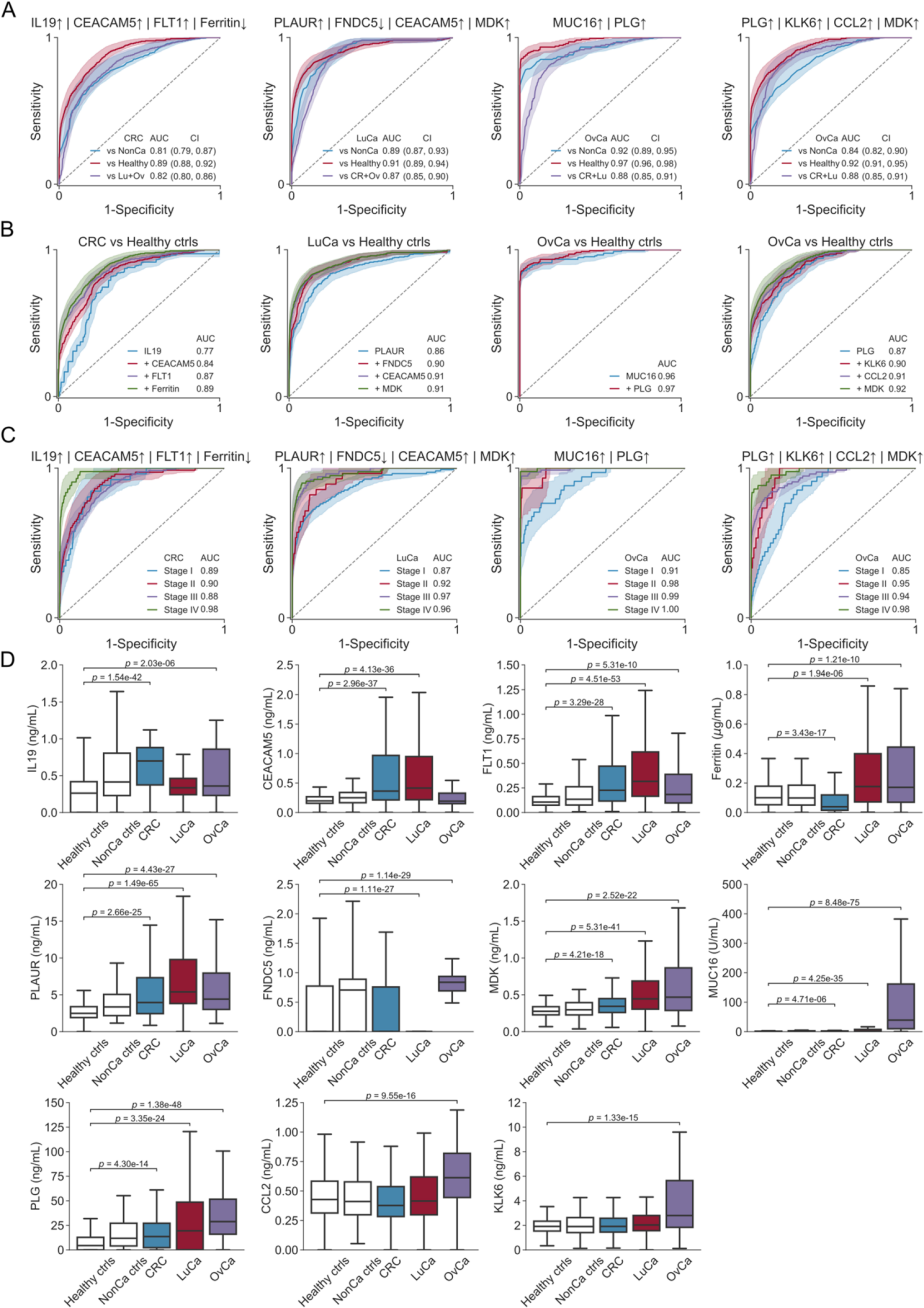
Composite biomarkers distinguish patients with either CRC, LuCa or OvCa from healthy individuals and from patients with the two other forms of cancer. The ROC was computed for all combinations of 1–4 of the selected analytes for each cancer type. For each combination evaluated, we optimized the ROC curve by exhaustive search on all possible cutoff values combined either by logical OR (|) or logical AND (&). The resulting composite biomarker was evaluated for superiority with respect to Epi proColon (AUC 0.82), FIT (AUC 0.88) and Cologuard (AUC 0.93) at the 99% confidence level using Bonferroni correction for multiple hypothesis testing. **A** ROC plots with AUC values for the top composite biomarkers for colorectal cancer (CRC), lung cancer (LuCa), ovarian cancer (OvCa), and OvCa when CA125/MUC16 was excluded from computation of combinations. **B** Sequential addition of analytes reveals their contribution to the ROC performance of the composite biomarkers. **C** Stage-dependent performance of the composite biomarkers over stages I-IV. **D** Boxplots of measured plasma concentrations for the analytes used in the composite biomarkers

For each tumor type, all possible composite biomarkers combining 2, 3 and 4 analytes were checked for superiority against these three predicate device tests (Supplementary Fig. 6). For CRC, only 2 composite biomarkers of 4 analytes were significantly superior to a test on par with Epi proColon, while for LuCa, there were 3 composite biomarkers of 2 analytes, 60 of 3 analytes, and 783 of 4 analytes that met the benchmark. For OvCa many composite biomarkers were significantly better than a test with performance on par with Cologuard (Table 3, Supplementary Tables 10–17). For the best biomarkers per tumor type, a combination of upregulated CEACAM5 (also known as CEA), FLT1 and IL19, and downregulated Ferritin separated CRC from healthy population controls (AUC 0.89), and a combination of downregulated FNDC5 and upregulated MDK, PLAUR and CEACAM5 separated LuCa from healthy population controls (AUC 0.91) (Fig. 3B). Both these biomarkers were statistically significantly superior to a biomarker on par with the CRC blood test Epi proColon but not to a biomarker on par with FIT. A combination of upregulated MUC16 (CA125) and PLG separated OvCa from healthy population controls (AUC 0.97) with performance significantly superior to a test with performance on par with Cologuard. Excluding CA125, which performed exceptionally well here as compared to literature, a combination of upregulated PLG, KLK6, MDK and CCL2 separated OvCa from healthy population controls with AUC 0.92, which was significantly superior to a biomarker with performance on par with Epi proColon. This performance is comparable to that of the commercially available test OVA1, consisting of five biomarkers: CA125, transthyretin, apolipprotein A1, beta-2 microglobulin, and transferrin [55], albeit OVA1 is not approved for screening. The best biomarkers for each tumor type could also separate cancer cases from the non-cancer controls with AUC 0.81–0.92 and discriminate the target tumor type from the two other tumor types with AUC 0.82–0.88 (Fig. 3A). Closer analysis of the best biomarkers per tumor type revealed that all the individual analytes contributed to the performance of the respective composite biomarker (Fig. 3B). Further, the composite biomarkers discriminated later stage tumors from healthy controls better than they discriminated earlier stages from healthy controls (Fig. 3C), which was also the case for the individual analytes included in the combinations. All of the top-performing composite biomarkers were determined from plasma protein levels only (Fig. 3D), none included metabolites. Replacing upregulated MDK protein with upregulated metabolite S-LDL-PL % in the four-combination for LuCa resulted in slightly worse but comparable ROC AUC (Supplemental Table S13) but overall, the benefit of adding a fourth analyte to the three-protein core of PLAUR, FNDC5 and CEACAM5 was small. Similarly, the metabolite GlycA was included in significant OvCa biomarkers but only as marginal improvements to a strong core based on protein combinations. Several proteins contributing to top-performing composite biomarkers, including FLT1, IL19, FNDC5, and PLG, were not measured in CancerSeek and therefore could not be evaluated in that dataset. These markers should therefore be interpreted as novel candidate biomarkers whose relevance remains to be established in future independent cohorts with harmonized proteomic coverage. In summary, we discovered two-protein biomarkers for OvCa and four-protein biomarkers for CRC and LuCa that were comparable to relevant benchmark FDA approved blood and/or stool tests for detection of cancer.

**Table 3:** Single analyte and composite plasma biomarkers for diagnosis of cancer compared to the ROC AUC of different benchmarks

Criterium^a^	CRC	LuCa	OvCa
Number of analytes	10	22	13
Superior to biomarker with			
AUC 0.82	0	0	1
AUC 0.88	0	0	1
AUC 0.93	0	0	0
Evaluated combinations of 2	90	462	156
Superior to biomarker with			
AUC 0.82	0	3	29
AUC 0.88	0	0	24
AUC 0.93	0	0	2
Evaluated combinations of 3	240	3080	572
Superior to biomarker with			
AUC 0.82	0	60	156
AUC 0.88	0	0	132
AUC 0.93	0	0	19
Evaluated combinations of 4	420	14,630	1430
Superior to biomarker with			
AUC 0.82	2	783	523
AUC 0.88	0	0	440
AUC 0.93	0	0	37

^a^The optimal ROC AUC was computed for all combinations of 2, 3 or 4 significantly altered analytes per tumor type (10 for CRC, 22 for LuCa, 13 for OvCa). For a combination of *k* analytes we computed 2^*k + 1*^ classification scenarios: each analyte could be up- or downregulated and the analytes combined using logical AND or logical OR. For each classification scenario exhaustive search is used to check all possible cutoff values and optimize the ROC curve, i.e. finding highest possible true positive rate for every false positive rate yielding the highest possible AUC. Each combination was checked for superiority at the 99% confidence level (Bonferroni correction) to biomarkers on par with the predicate devices Epi proColon (AUC 0.82), FIT (AUC 0.88) and ColoGuard (AUC 0.93)

### Tumor-stage-dependent biomarkers

**Fig. 4:**
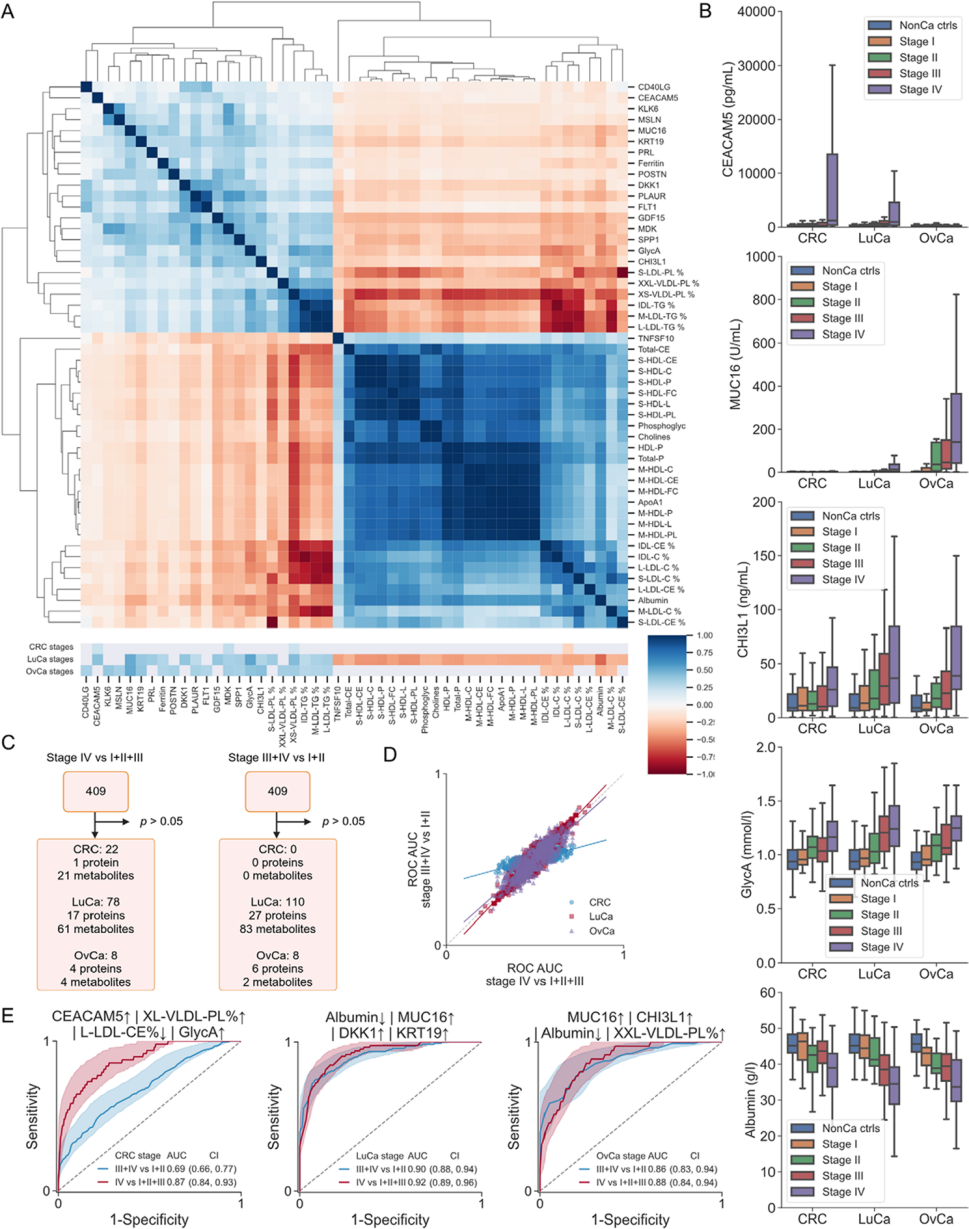
Tumor-stage-dependent proteomic and metabolomic signatures. **A** Clustered heatmap of Spearman rank correlations between analytes and stages for CRC, LuCa and OvCa, respectively. Lower heatmap displays for which type of cancer the analytes had significant correlation with stage. Significant correlations with stage were observed for 4 proteins in CRC, 111 in LuCa and 23 in OvCa (adjusted *p* < 0.05). **B** Boxplots of analyte concentrations for different tumor types and stages. **C** Identification of analytes of all 409 (165 proteins and 244 metabolites) measured that could separate stage IV from stages I-III using the Mann-Whitney *U* test as well as analytes that could separate stages III-IV from stages I-II. **D** ROC AUC values for separating stage IV from stages I-III, plotted against ROC AUC values for separating stage III-IV from stages I-II for all 409 measured single analytes. **E** The ROC for the top combinations of composite biomarkers separating stages III-IV from stages I-II as well as separating stage IV from stages I-III. For each combination of selected analytes evaluated, we optimized the ROC curve by exhaustive search on all possible cutoff values combined either by logical OR (|) or logical AND (&)

We also wanted to identify biomarkers that could discriminate cancer stage. We therefore evaluated correlations between protein and metabolite concentrations and stage for CRC, LuCa and OvCa. We analyzed the 818 cancer cases and 119 non-cancer controls by Spearman rank correlation test, including all measured proteins and metabolites since the pre-analytical effects from the healthy control cohort comparisons are not an issue here. We found 4 (2 proteins, 2 metabolites) analytes for CRC, 111 (27 proteins, 84 metabolites) analytes for LuCa and 23 (12 proteins, 11 metabolites) analytes for OvCa that significantly correlated with stage (95% confidence level, Bonferroni correction), with the MDK protein, and the two metabolites XS-VLDL-PL % and L-LDL-C % overlapping for all the three tumor types (Fig. 4A-B, Supplementary Table 18). Next, we searched for single analytes that could discriminate stages III-IV from stages I-II tumors with statistical significance by computing ROC AUC, and found 0 analytes for CRC, 110 analytes (27 proteins, 83 metabolites) for LuCa and 8 analytes (4 proteins, 4 metabolites) for OvCa (2-sided Mann-Whitney *U* test under Bonferroni correction; Fig. 4C-D; Supplementary Table 19). In the same way, we also searched for single analytes that could distinguish stage IV from stages I-III and found 22 (1 protein, 21 metabolites) for CRC, 78 (17 proteins, 61 metabolites) for LuCa, and 8 (4 proteins, 4 metabolites) for OvCa (Supplementary Fig. 6). From the single analytes that could separate stage IV cancers from lower stages, we computed all possible 4-combination composite biomarkers and the top combinations included upregulated CEACAM5, XL-VLDL-PL %, GlycA and downregulated L-LDL-CE % for CRC (ROC AUC 0.87), upregulated MUC16, DKK, KRT19 and downregulated albumin for LuCa (ROC AUC 0.92), and upregulated MUC16, XXL-VLDL-PL %, CHI3L1 and downregulated albumin for OvCa (ROC AUC 0.88) (Fig. 4E, Supplementary Tables 20–22). Downregulated albumin in LuCa and OvCa is consistent with the hypoalbuminemia commonly observed in wide-spread cancer. The discrimination of stage IV CRC from stage I-III CRC was, as could be expected, much better compared to the discrimination of stages III-IV from stage I-II. This is in contrast to LuCa and OvCa, where there was similar performance for stage IV vs. I-III and stage III-IV vs. I-II (Fig. 4D). This is however consistent with our earlier observation that no single analytes were statistically significant for separation of CRC stage III + IV from stage I + II. In summary, blood-based resolution of tumor stage based on the proteins and metabolites measured was more effective for LuCa and OvCa than for CRC, and metabolites contributed to the performance of several of the best composite biomarkers.

### External validation of proteomic biomarkers

**Fig. 5:**
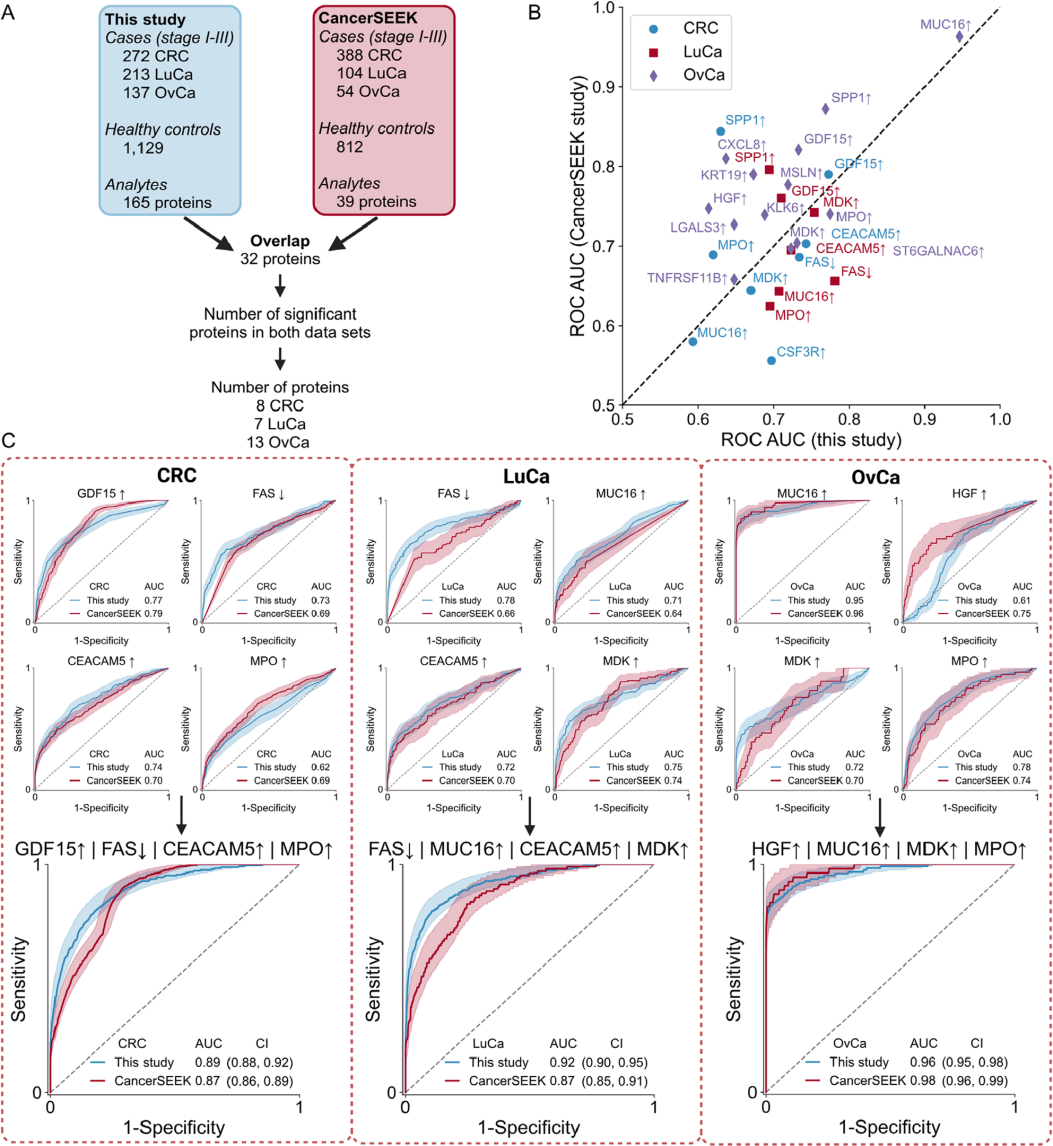
Validation of proteomic biomarkers for cancer detection in an external dataset. For a subset of proteins, external validation could be performed in CancerSEEK data. **A** Cohort sizes and overlap in measured proteins in this study and in the CancerSEEK study. In total, 32 proteins were measured in both studies and 3 of these were excluded due to technical issues. Next, we enriched for proteins with consistent behavior, selecting for each tumor type those with ROC AUC > 0.5 for cancer stage I-III vs. healthy controls in both studies. This resulted in 8 CRC, 7 LuCa and 13 OvCa proteins considered informative in both studies. **B** The ROC AUC in this study (discovery) plotted against the ROC AUC in CancerSEEK study (validation) for all proteins regarded informative after filtering. **C** The best 4-combination biomarkers of informative proteins found in this study for CRC, LuCa and OvCa, respectively, and the corresponding performance in CancerSEEK. For each biomarker, the ROC curves and AUC of the individual proteins and the composite biomarker are shown

Finally, we performed external validation of individual proteins and selected composite biomarkers for detection of cancer vs. healthy controls (Fig. 5, Supplementary Fig. 6). To this end, we compared the data from stage I-III cancers and healthy controls in this study to data from the CancerSeek study [11] where Luminex technology was used to measure one-quarter of the proteins analyzed here (Fig. 5A). We found several single-protein biomarkers, including MUC-16/CA125 and GDF15, that had highly similar ROC AUC values between our study and the CancerSeek study, whereas some were slightly superior in CancerSeek (SPP1/OPN) or in this study (PECAM-1), respectively (Fig. 5B, Supplementary Table 23). In all, 32 proteins were measured in both studies and after filtering analytes with technical issues we selected proteins with consistent behavior for each diagnosis. When 4-protein composite biomarkers were generated using the 8 retained proteins for CRC, 7 retained proteins for LuCa and 13 retained proteins for OvCa, similar ROC performance was achieved in both datasets (Fig. 5C, Supplementary Tables 24–26). In conclusion, we observed a high degree of similarity for biomarkers that we sought to validate in an independent data set, indicating that composite protein biomarkers can form the bases of robust and reproducible diagnostic tests for cancer detection.

## Discussion

Here, we analyzed a large dataset and identified combinations of 2–4 analytes/proteins for all three tumor types with ROC AUC performance superior to an FDA-approved early cancer detection blood test. Based on FDA pre-approval decisions on biomarkers since 1981, the Epi proColon ROC performance of 0.82 can be considered as a bar to surpass for a new blood-based biomarker test for early detection of cancer to have potential for regulatory approval and wider clinical adoption. The identified composite plasma all-protein biomarkers that exceed the selected benchmark merit further development and evaluation in cohorts collected in a healthcare triage or screening setting. In both CRC and LuCa we found biomarkers that were significantly superior to the performance of detection of CRC by Epi proColon. In OvCa, we identified biomarkers with ROC performance superior to the at the time of analyses most sensitive FDA approved early detection test for cancer, which was the feces test Cologuard with ROC AUC 0.93 for detection of CRC. The OVA-1 test is an FDA-approved blood serum test for assessing risk for ovarian malignancy in women previously diagnosed with an ovarian mass for which they are scheduled for surgery. However, OVA1 is not a test for early cancer detection in women that do not have an already detected ovarian mass and is therefore not a suitable benchmark for OvCa in this study. During 2024, past the analyses of this study, two new early detection tests for CRC obtained FDA premarket approval, the cfDNA blood-based Shield test, and the multitarget stool DNA test Cologuard Plus, and submission is underway for a blood-ctDNA test from Freenome. In clinical evaluation, the Shield test had 83.1% sensitivity [56], and Cologuard Plus 93.9% sensitivity [57] for detection of CRC, both higher than the corresponding benchmark tests used here. Although the new blood-based tests outperform the previous state-of-art, the Epi proColon test, detection of early stage cancer and precancerous lesions remains a challenge, with sensitivity of the Shield and Freenome tests for clinical stage I cancer being 55–57% and for advanced precancerous lesions 13-15.5% [17, 56].

To the best of our knowledge, this is the first study integrating affinity proteomics and metabolomics to develop composite biomarkers. The cores of the composite biomarkers identified here contained well-established single protein biomarkers such as MUC16/CA125 or CEACAM5/CEA, effectively supporting their relevance. In CRC, while FLT1 has previously been found elevated [58], IL-19 has not. Elevated PLAUR/suPAR is previously reported in non-small cell LuCa (NSCLC) but not in small cell LuCa (SCLC) [59], whereas elevated FNDC5/irisin has not previously been linked to LuCa. Consistent with our data, elevated Midkine (MDK) has previously been found in LuCa [60] and OvCa [61]. An interesting observation is that, as compared to the literature, MUC16/CA125 performed extremely well for detection of OvCa in our data, and also in the external Luminex-based validation dataset from the CancerSeek study. While CA125 is in use in OvCa monitoring and prognostics, including as part of the OVA1 test [62, 63], it has some issues that limit its clinical value. For example, it was previously found not to be elevated in ~ 20% of women with epithelial OvCa [64]. One potential explanation is that the antibodies to CA125 used in the Luminex assays perform better than those used in other affinity proteomics assays, which could potentially be utilized to improve current CA125-containing biomarkers. Interestingly, plasminogen (PLG) elevation, which has not previously been reported in OvCa, was part of strong biomarkers in combinations both with and without CA125. In contrast, both of the two other elevated plasma proteins in the best OvCa biomarker without CA125, KLK6 and CCL2, have prior association with OvCa [65, 66]. For NMR metabolomics, this study encompasses twice the number of CRC cases [67, 68], twice the number of LuCa cases [69] and twice the number of OvCa cases [70] than prior art. Several NMR analytes of interest here, including albumin and GlycA, have regulatory approval for clinical use, which could enable implementation of composite biomarkers incorporating metabolites. However, the cohort differences impacted variability for metabolites more than was the case for proteins in this study, potentially because of differences in fasting status before blood draw [71] between the two cohorts. We found potentially useful metabolites for stage determination when only the U-CAN cohort was analyzed. The observation that only two metabolites passed the pre-analytical filtering when U-CAN cases were compared to the healthy controls suggests that NMR-based tests will require highly standardized sampling and handling for robustness of clinical implementations. In summary, the best performing composite biomarkers for each tumor type combine known and novel biomarkers, supporting their relevance.

Interpretation of these results should consider several potential confounders. Pre-analytical differences between cohorts, including fasting status and sample handling, may affect circulating protein and metabolite levels, particularly in comparisons involving population-based healthy controls. In addition, lifestyle variables such as smoking status, especially relevant for lung cancer, were not uniformly available and could not be fully controlled for in healthy controls. The study populations were also derived predominantly from Northern European cohorts, and population-level differences related to genetic background, environmental exposures, and healthcare context may influence biomarker distributions and predictive performance. To mitigate these effects, analyte selection was anchored to cancer versus non-cancer comparisons within the same biobank, and analytes showing strong discrimination between non-cancer and healthy controls were excluded prior to composite biomarker construction. While this conservative strategy reduces cohort, lifestyle and population-related bias, residual confounding cannot be excluded. Accordingly, the identified biomarkers should be viewed as robust candidates under the applied constraints rather than definitive disease-specific signatures, and future studies with harmonized sampling, matched lifestyle variables, and validation across diverse populations will be required before their clinical application.

Another potential use of cancer biomarkers is to separate early from late-stage tumors. Interestingly, a larger number of metabolites compared to proteins had statistically significant differences between early- and late-stage cancer cases. Some analytes, such as albumin and GlycA, are well-known general disease markers and are likely not tumor specific. However, in combination with more tumor-specific proteins this could still be useful in a composite biomarker. For LuCa and OvCa, many analytes showed consistent increase or decrease with increasing stage (correlation analysis and box plots in Fig. 4), and discriminated stages III-IV from stages I-II with similar ROC AUC as discriminating stage IV from stages I-III. This was not the case for CRC, where most analytes displayed a large difference between stage IV and stages I-III (Fig. 3C). This is consistent with the generally lower performance for CRC biomarkers compared to LuCa and OvCa discovered in this study. Overall, metabolites proved more useful for tumor stage discrimination than they did for early detection.

Emerging biomarker modalities such as circulating tumor DNA and quantitative transcriptomics offer complementary strengths to proteomics or metabolomics. While genomic assays provide high specificity for tumor-derived alterations, their sensitivity in early-stage disease may be limited by low analyte abundance. Protein biomarkers capture both tumor- and host-response signals and may be detectable earlier. The ROC-based framework used here is modality-agnostic and can be extended to genomic or integrated multi-modal datasets with appropriate quantitative measurements and harmonized study design.

Here, we used large numbers of samples and a novel computational approach [29] to evaluate combinations of analytes, for each combination identifying the optimal direction of change for each analyte that produced the best ROC AUC performance. While partitioning of data for training, test and validation is current convention, we reasoned that external validation by analysis of independently generated data is more meaningful given that study-specific biases caused by pre-analytical sample handling and cohort composition are not corrected for by partitioning. After removing analytes with inflated ROC AUC likely due to pre-analytical cohort differences, we used hypothesis testing under Bonferroni correction to determine if the combinations superseded clinical benchmarks tests in diagnostic performance. While a potential drawback of the filtering approach applied is the loss of biomarkers that also recognize non-malignant pre-cancerous lesions, we reasoned that it would be more useful to unambiguously identify biomarkers of malignant disease. While the approach used here identifies the combinations of analytes giving the best ROC performance, the optimal cut-off values need to be determined through analysis of additional data. To provide true external validation, we will next analyze the identified composite biomarkers in samples collected before diagnosis at suspicion of cancer as well as in population screening setting, which represents the main use for diagnostic blood biomarkers. A potential use of biomarkers with high tumor-type specificity, as the ones identified here, is to discriminate abdominal tumor types when patients present with diffuse symptoms that are not disease-specific. The discrimination of OvCa from CRC, where patients with disseminated OvCa may present with similar symptoms and radiological findings as CRC patients, exemplifies such clinical needs where a reliable blood test could be a supplement or even alternative for biopsy if validated clinically.

Since infrastructure and workflows are straightforward, cost effective and well established in clinical laboratories, protein-based diagnostic biomarkers for cancer may be preferred as compared to analyses of ctDNA or ctRNA. However, the requirements for sensitivity and tumor type specificity are rarely fulfilled by single-analyte biomarkers, but demand a combination of several proteins, metabolites or nucleic acid targets [72]. For reliability and cost reasons, a diagnostic test based on such combinations should incorporate as few analytes as possible. We therefore capped combination sizes at 4 analytes in this study. Although this is a low number considering other prominent proteomics-containing cancer signatures [11], we identified hundreds of potential tumor-type-specific biomarkers that perform at least equally well as the prior art. While there are other affinity proteomics technologies that enable measurement of thousands of proteins and can thus provide much larger datasets for de novo biomarker discovery, the one used here is implemented on an FDA approved instrument platform, which can facilitate clinical translation. The biomarkers in this study could either be shown to behave similarly versus non-malignant controls from both study cohorts, or be validated in an external cohort. However, as we measured 4-fold more proteins than the external dataset from the CancerSeek study, only a subset of combinations could be externally validated at this point. The present study concerns the biomarker discovery stage, using retrospective case–control cohorts to prioritize candidate biomarkers under strict statistical control. Performance metrics from enriched case-control cohorts are not directly comparable to those from prospective screening studies and should not be extrapolated to asymptomatic populations or pre-malignant lesions. Future studies will require substantially larger cohorts due to the low prevalence of early-stage disease or asymptomatic patients in the unselected cohorts. Accordingly, comparisons to established screening tests are intended as discovery-stage benchmarks, and prospective population-scale studies will be required to assess true screening performance and clinical utility. Several of the proteins we could compare had remarkably similar ROC AUC for cancer versus population controls in CancerSEEK, while others were slightly different. The observed differences can indicate sensitivity to pre-analytical variation or be attributed to the different age distributions of cases and controls in the respective cohorts. All of this elevates the importance of pre-analytical filtering or the robust assessment of the biomarkers in various study populations, to promote only the best analytes for clinical application.

## Supplementary Information

Supplementary Material 1.

Supplementary Material 2.

## Data Availability

The datasets generated in the study for cases and non-cancer controls from the U-CAN cohort are available in the SciLifeLab Data Repository, with DOI: 10.17044/scilifelab.28351367.v2. Datasets generated for EpiHealth healthy controls are not publicly available due to limitations with the informed consent, however, are available from the corresponding author on reasonable request.
